# Cold‐induced beigeing of stem cell‐derived adipocytes is not fully reversible after return to normothermia

**DOI:** 10.1111/jcmm.15749

**Published:** 2020-09-09

**Authors:** Hilda Anaid Lugo Leija, Ksenija Velickovic, Ian Bloor, Harold Sacks, Michael E. Symonds, Virginie Sottile

**Affiliations:** ^1^ Wolfson STEM Centre School of Medicine The University of Nottingham Nottingham UK; ^2^ The Early Life Research Unit Division of Child Health, Obstetrics and Gynaecology The University of Nottingham Nottingham UK; ^3^ VA Endocrinology and Diabetes Division Department of Medicine University of California Los Angeles CA USA; ^4^ Nottingham Digestive Disease Centre and Biomedical Research Centre School of Medicine The University of Nottingham Nottingham UK; ^5^ Department of Molecular Medicine The University of Pavia Pavia Italy

**Keywords:** adipogenesis, brown adipose tissue, differentiation, stem cell, temperature

## Abstract

Beige adipocytes possess the morphological and biochemical characteristics of brown adipocytes, including the mitochondrial uncoupling protein (UCP)1. Mesenchymal stem cells (MSCs) are somatic multipotent progenitors which differentiate into lipid‐laden adipocytes. Induction of MSC adipogenesis under hypothermic culture conditions (ie 32°C) promotes the appearance of a beige adipogenic phenotype, but the stability of this phenotypic switch after cells are returned to normothermic conditions of 37°C has not been fully examined. Here, cells transferred from 32°C to 37°C retained their multilocular beige‐like morphology and exhibited an intermediate gene expression profile, with both beige‐like and white adipocyte characteristics while maintaining UCP1 protein expression. Metabolic profile analysis indicated that the bioenergetic status of cells initially differentiated at 32°C adapted post‐transfer to 37°C, showing an increase in mitochondrial respiration and glycolysis. The ability of the transferred cells to respond under stress conditions (eg carbonyl cyanide‐4‐phenylhydrazone (FCCP) treatment) demonstrated higher functional capacity of enzymes involved in the electron transport chain and capability to supply substrate to the mitochondria. Overall, MSC‐derived adipocytes incubated at 32°C were able to remain metabolically active and retain brown‐like features after 3 weeks of acclimatization at 37°C, indicating these phenotypic characteristics acquired in response to environmental conditions are not fully reversible.

## 1 INTRODUCTION

Brown and white adipocytes have different origins and functions.^1^ White adipocytes, which provide energy storage in the form of triglycerides, are characterized by single large lipid droplets and few mitochondria. By contrast, brown adipocytes are multilocular and contain numerous small lipid droplets and mitochondria that promote energy expenditure through heat production.^1^, ^2^, ^3^, ^4^ A unique characteristic of brown adipocytes is the presence of the mitochondrial uncoupling protein 1 (UCP1), which when stimulated enables the free flow of protons across the inner mitochondrial membrane without phosphorylating ADP,^2^, ^5^ conferring a high potential thermogenic capacity to brown adipose tissue.^6^ In addition to brown and white adipocytes, beige adipocytes have been reported to display a similar morphology to white cells in the basal state, but can be stimulated by cold exposure to acquire an activated brown‐like morphology with significant UCP1 expression.^7^, ^8^, ^9^, ^10^ The extent to which temperature‐induced stimulation of beige fat can be maintained upon return to a warm environment is unclear. In vivo, it appears that beige adipocytes from cold‐stimulated mice revert to white fat within six weeks of warm acclimatization.^11^ Similarly, in vitro differentiation of brown‐like adipocytes mediated by culture at 27‐33°C for 10 days was not maintained after the temperature was restored to 37°C.^7^ Once established, however, classical brown adipocytes have been reported to maintain their multilocular morphology for one month in the same culture conditions.^12^ This longer term adaptation appears to be mediated by a different mechanism from promoting the initial appearance of UCP1 and could include adaptations in the rate of biogenesis^12^ and mitophagy.^13^ Mesenchymal stem cells (MSCs), which are progenitors of adipocytes among other lineages,^9^, ^14^, ^15^, ^16^, ^17^, ^18^ can respond to cold exposure during in vitro differentiation by forming UCP1‐expressing adipocytes,^10^ but the extent to which this induced phenotype can be retained after returning to normothermic conditions (ie 37°C) is not known. The present study therefore examined the stability of the acquired cellular features using an in vitro model of MSC differentiation under hypothermic conditions and demonstrates that these differentiated cells maintained some beige‐like features and an intermediate thermogenic phenotype over 3 weeks of subsequent culture under a temperature of 37°C.

## 2 MATERIALS AND METHODS

All reagents were purchased from Thermo Fisher Scientific (Loughborough, UK) unless otherwise stated.

### 2.1 Cell culture and differentiation

**FIGURE 1:**
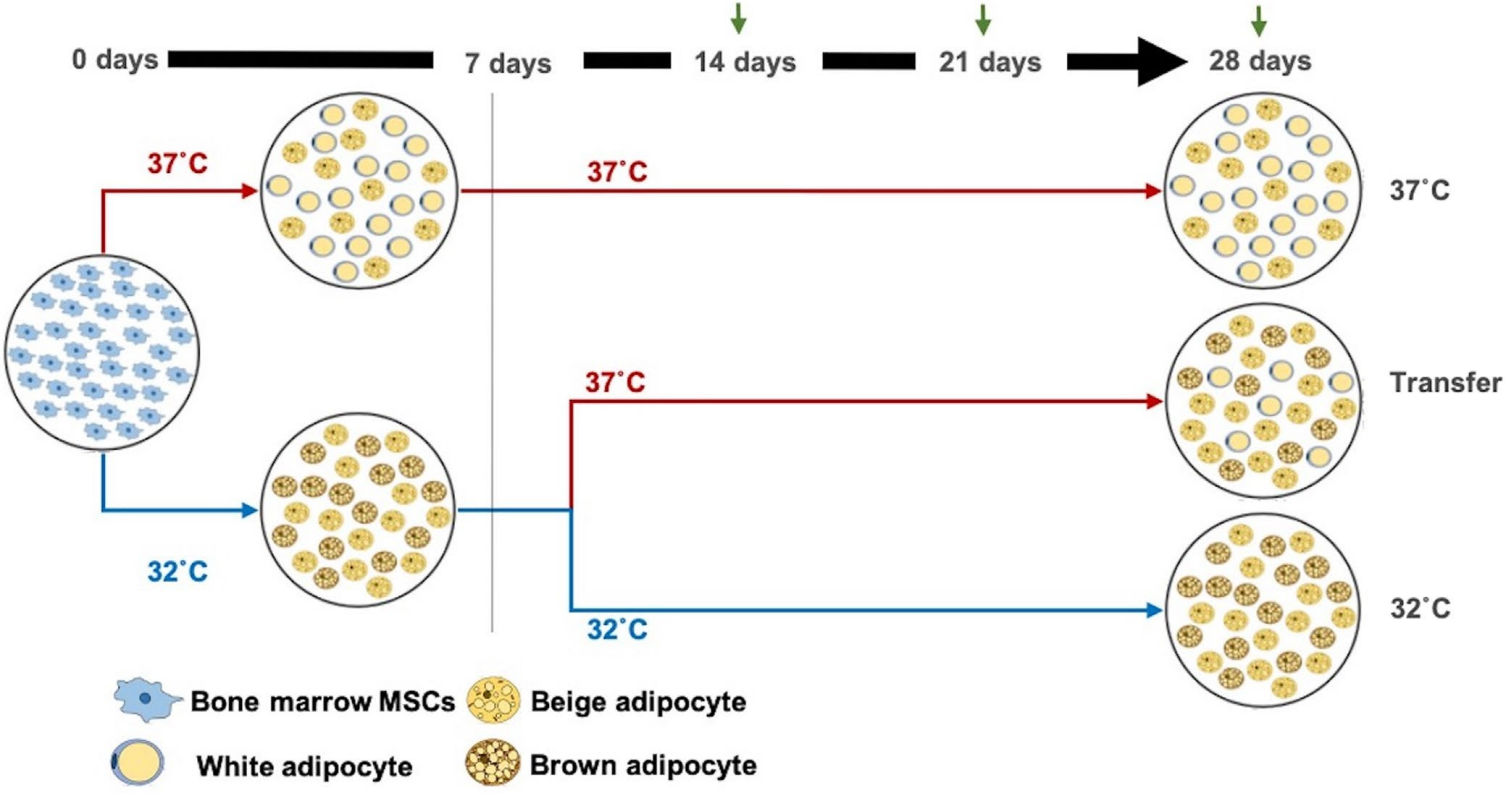
Schematic diagram of MSCs adipogenesis treatments. MSCs were cultured in adipogenic medium at 37°C or 32°C for 7 days and then either maintained at this temperature or transferred to 37°C. Green arrows indicate samples’ time‐points at 14, 21 or 28 days

Mouse MSC cells (MSCs)^10^ were cultured in standard medium (SM) made with DMEM low‐glucose supplemented with 10% foetal bovine serum, 2 mM l‐Glutamine, 1% NEAA (non‐essential amino acids) and 1% penicillin/streptomycin. For adipogenic differentiation, cells at 90% confluency were treated with adipogenic medium (AM) prepared by supplementing SM with 10 µg/mL insulin (Sigma‐Aldrich, Gillingham, UK), 1 µM rosiglitazone (Cayman Chemicals, Ann Arbor, MI, USA), 1 µM dexamethasone (Cayman Chemicals), 100 µM IBMX (Sigma‐Aldrich) and 1nM triiodothyronine (T3) (Sigma‐Aldrich) for up to 28 days. In order to evaluate the effect of incubation temperature on the morphology, function and molecular signature of MSCs, three different temperature conditions were used during cell differentiation (Figure 1), that is 37°C or 32°C in 5% CO_2_ throughout, or 32°C for 7 days and then transferred to 37°C (referred to as ‘Transfer’). The culture medium was changed every 2‐3 days, and cells were sampled at 14, 21 or 28 days for analysis.

### 2.2 Metabolic activity assay

Presto Blue Cell Viability Reagent was used as previously described^10^ to measure metabolic activity at day 14, 21 and 28. Fluorescence measurements were taken on 100 µL triplicate samples using a multimode microplate reader (Tecan Infinite 200 PRO, Tecan, Switzerland) using an excitation and emission wavelengths of 560 nm and 590 nm, respectively.

### 2.3 Oil Red O (ORO) imaging and quantification

Fixed cells were stained with ORO as previously described.^10^ The staining intensity was quantified by extracting ORO staining with 100% isopropanol, before measuring absorbance in triplicates at 510 nm with a multimode microplate reader (Tecan Infinite 200 PRO, Tecan, Switzerland).

### 2.4 Mitochondrial staining

Live cells were stained for 45 min with MitoTracker Deep Red (100 nM) and Hoechst 33 258 (20 μg/mL), according to the manufacturer's protocol, before fixation. Samples were mounted with FluoroGel mounting medium (GeneTex, Irvine, CA, USA) and analysed with a Zeiss Elyra PS.1 microscope (Cambridge, UK). Mitochondria numbers were evaluated using a total of 15 randomly selected images per condition of three biological replicates. Relative fluorescence intensities were measured using the ImageJ software (https://imagej.nih.gov/ij/).

### 2.5 Calcium staining and quantification

Live cells were stained for 60 min with Fluo‐4 Direct^TM^ (Invitrogen, Loughborough, UK) according to the manufacturer's instructions. Briefly, a final volume of 1X Fluo‐4 Direct^TM^ calcium reagent was added to each well and incubated at 37°C or 32°C. Fluorescent measurements were performed in triplicates using an excitation wavelength of 494 nm and an emission wavelength of 516 nm with a multimode microplate reader (Tecan Infinite 200 PRO, Tecan, Switzerland). After the measurement, live fluorescent images were taken using a Zeiss Elyra PS.1 microscope.

### 2.6 Immunocytochemistry

UCP1 immunocytochemistry was performed as previously described,^10^ using a 1:500 solution of anti‐UCP1 antibody (Abcam, Cambridge, UK) and a 1:500 dilution of Alexa‐633‐conjugated secondary antibody. Samples were mounted with FluoroGel mounting medium and examined with a Zeiss Elyra PS.1 microscope. Relative fluorescence intensities were measured with ImageJ software (https://imagej.nih.gov/ij/).

### 2.7 Gene expression analysis

Total RNA was extracted, diluted to 1 µg/µL and reverse transcribed to cDNA as previously described.^10^, ^19^ Expression levels for UCP1, PR/SET Domain 16 (PRDM16), Cytochrome C Oxidase Subunit 8B (COX8β), Purinergic Receptor P2X 5 (P2RX5), Iodothyronine Deiodinase 2 (DIO2), Transient Receptor Potential Cation Channel Subfamily V Member 1, 2 and 4 (TRPV1, TRPV2 and TRPV4, respectively) were determined using TaqMan probes (Thermo Scientific TaqMan Gene Expression assays; assay: Mm00494069_m1, Mm00712556_m1; and BioRad TaqMan Gene Expression assays; assay, qMmuCIP0034367, qRnoCIP0024301, qMmuCEP0052679, qMmuCIP0031313, qMmuCIP0035343 and qMmuCIP0032629, respectively). Primers used in this study were previously described.^10^ Gene expression was determined using the GeNorm normalization algorithm against two selected reference genes (stability value M = 0.36), *B2M* (Beta‐2‐Microglobulin) and acidic ribosomal protein subunit P0 (RPLP0). Reference genes are shown in Table 1.

**TABLE 1:** List of primers used for gene expression analysis

**Target gene**	**Forward Primer (5’‐> 3´)**	**Reverse Primer (5´‐> 3´)**
RPLP0 (Ref Gene)	CTGGAGAAACTGCTGCCTCA	AGTGTTCTGAGCTGGCACA
B2M (Ref Gene)	CCCTGGTCTTTCTGG	TGTTCGGCTTCCCAT

### 2.8 Oxygen consumption analysis

Oxygen consumption was measured using a Seahorse XF96 Extracellular Flux Analyzer as previously outlined.^10^ Basal respiration rate, ATP‐linked respiration, proton leak respiration, maximal oxygen consumption rate (OCR), extracellular acidification rate (ECAR), mitochondrial reserve capacity and coupling efficiency were calculated as previously described.^10^, ^20^

### 2.9 Statistical analysis

Data analysis was performed using Kruskal‐Wallis test with Dunn's multiple comparisons test with the GraphPad Prism software. The changes in gene expression between each temperature and time‐point were reviewed using hierarchical clustering analysis (HCA) and principal component analysis (PCA) using ClustVis (https://biit.cs.ut.ee/clustvis/). Statistical significance was considered at *P < *.05, with *P < .05, **P < .01, ***P < .001. Graphs are presented as mean ± SEM (n = 3 individual experiments).

## 3 RESULTS

### 3.1 Cold‐induced adipocytes retain beige‐like cellular and morphological features when returned to a normothermic environment

**FIGURE 2:**
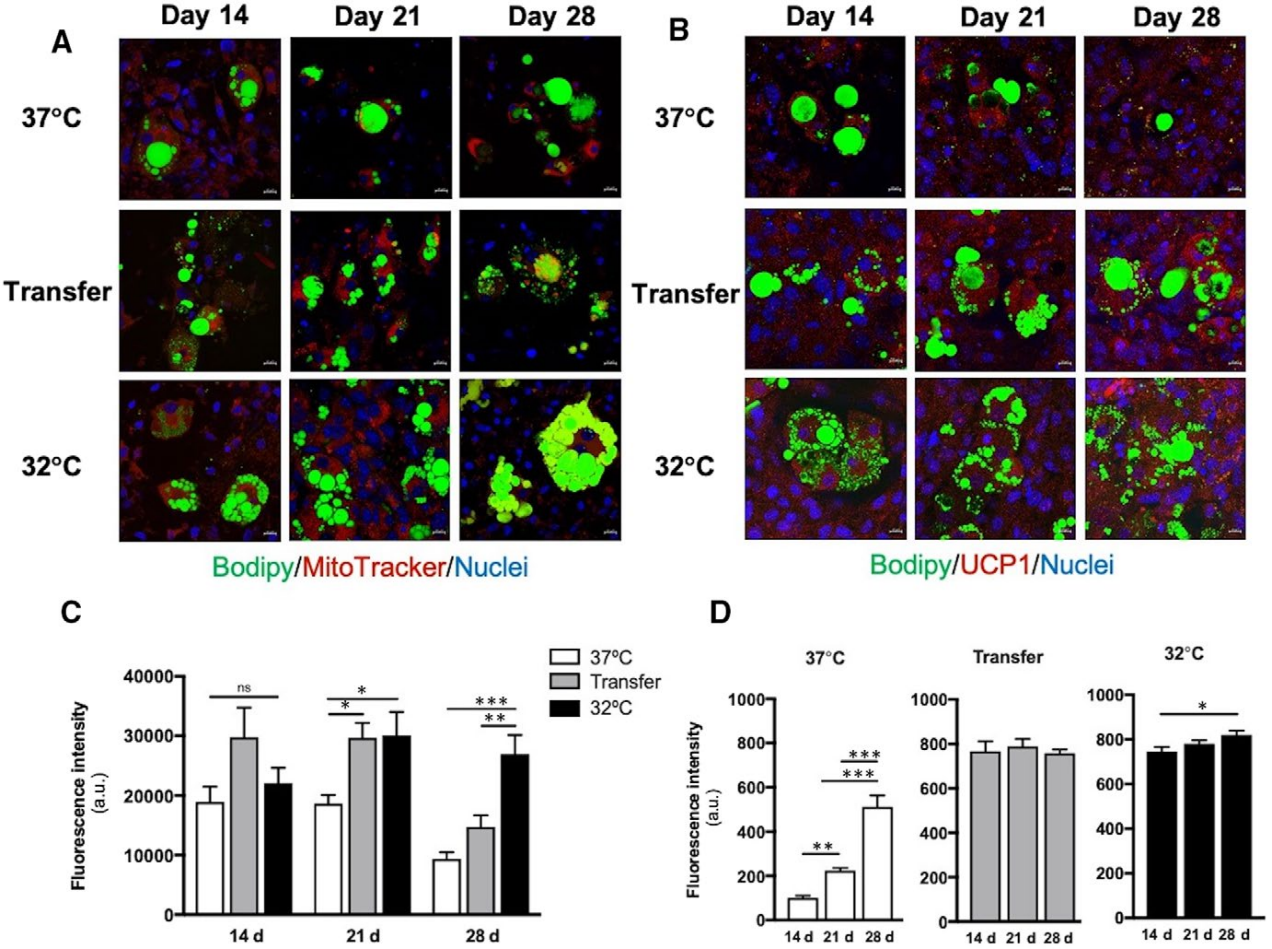
Effect of culture temperature on the abundance of mitochondria and uncoupling protein (UCP)1. Representative images of MitoTracker staining (A) and UCP1 (B) shown in red, with Hoechst 33 258 nuclear counterstain in blue and Bodipy in green. Scale bar = 10 µM. Relative fluorescence intensity of MitoTracker (C) and UCP1 (D) staining as measured in 15 randomly selected micrographs of 3 independent experiments. *P < .05, **P < .01, ***P < .001

To analyse the stability of the beigeing traits induced by hypothermic conditions, MSC were exposed to different culture environments (Figure 1) and their phenotypic, metabolic and molecular response was assessed over 28 days. When compared to normothermic control cultures, cells transferred from 32°C to 37°C showed similar expression of UCP1 protein and morphological features associated with hypothermic conditions, such as multilocular lipid droplets (LDs) and increased mitochondrial abundance which were maintained until day 21 (Figure 2).

**FIGURE 3:**
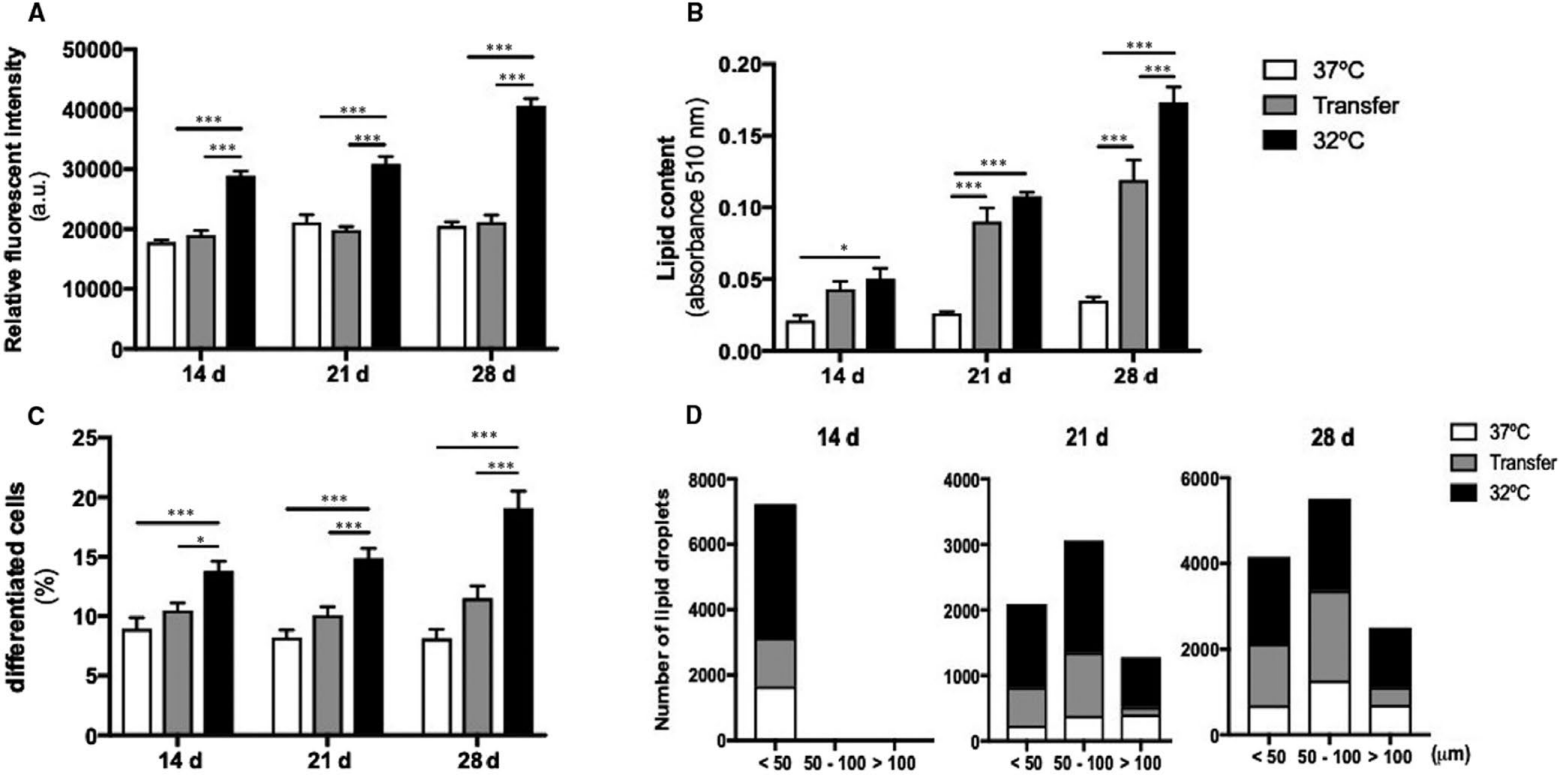
Cellular and morphological response of adipogenic MSCs differentiated under normal, Transfer and hypothermic conditions at 14, 21 and 28 days. (A) Metabolic activity measurements in adipogenic cultures. (B) Lipid content measured by Oil Red O staining in adipogenic cultures. (C) Proportion of differentiated cells containing lipid droplets. Data are shown as mean ± SEM. *P < .05, **P < .01, ***P < .001. (D) Total amount of lipid droplets’ size measured for each condition and time‐point, and lipid droplets > 50 μm were not detected on day 14. 15 micrographs were measured per condition, and size of lipid droplets was measured with Ferret's diameter (μm) and grouped according to their size

Compared with normothermic controls, metabolic activity was increased in cells maintained at 32°C, but not in cells subsequently transferred back to 37°C (Figure 3A). By contrast, lipid content increased over time in cells maintained at 32°C and in those subsequently transferred to 37°C (Figure 3B). The same response was observed when percentage of differentiated cells in each treatment (Figure 3C) and lipid droplet size (Figure 3D) were analysed. These changes in lipid content were reflected by differences in cell morphology, as MSCs differentiated at 37°C contained large perinuclear LDs consistent with a white phenotype, while those differentiated at 32°C were mostly multilocular with smaller LDs on days 14 and 21 and then exhibited larger lipid vacuoles by day 28. Cells transferred from 32°C to 37°C displayed mixed uni‐ and multilocular cells, which became more unilocular by 28 days (Figure S1).

### 3.2 Expression of beige and brown markers is maintained in cold‐induced adipocytes when returned to 37°C for 21 days

**FIGURE 4:**
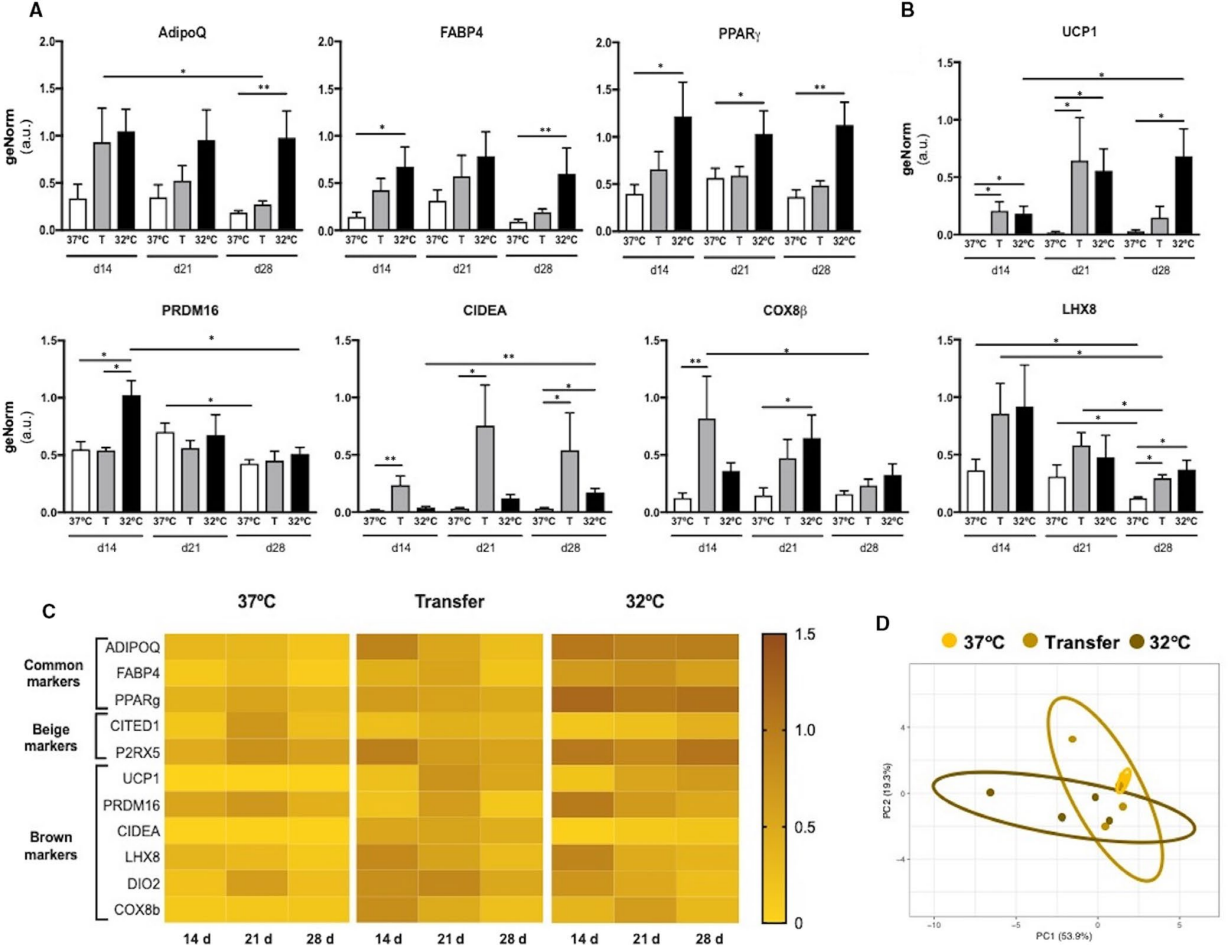
Effect of incubation temperature on gene expression. (A) Expression levels of common adipogenic markers: Adiponectin (ADIPOQ), FABP4 and PPARγ. (B) Expression level of brown adipogenic markers: UCP1, PRDM16, CIDEA, COX8β and LHX8. (C) Heat maps of the GeNorm normalization on expression levels of common adipogenic markers: Adiponectin, FABP4 and PPARγ, beige adipogenic markers: CITED1 and P2RX5, and brown adipogenic markers: UCP1, PRDM16, CIDEA, LHX8, DIO2 and COX8β. Each row represents a gene while each column symbolize a time‐point at 14, 21 and 28 days—brown = high (1.5), yellow = low (0). (D) Scatter plots of the two principal components containing 73.2% of total variance for day 28. n = 4 individual experiments per time‐point. Data represent the mean of four replicas ± SEM. Statistical significance was set at *P* < .05. *P < .05, **P < .01, ***P < .001

Gene expression changes analysed by real‐time PCR showed that while hypothermic conditions induced enhanced expression of adipogenic markers AdipoQ, FABP4 and PPARγ, expression in Transfer cells was not different from those differentiated at 37°C (Figure 4A). When evaluating brown markers, gene expression was higher in the hypothermic 32°C condition over the time‐course, compared with normothermic conditions, and Transfer cells showed higher UCP1 signal at day 14 and 21 which progressively decreased to 28 days, while CIDEA and PRDM16 gene expression was maintained over the time‐course. Expression of two other brown markers, COX8β and LHX8, also increased in Transfer cells compared with normal 37°C cultures, in a pattern comparable to that seen in hypothermic cultures (Figure 4B). By day 28, Transfer adipocytes exhibited a transitional molecular signature, between that of normothermic and hypothermic culture conditions (Figure 4C). Three brown and beige markers were also evaluated, that is DIO2, P2RX5 and CITED mRNA, but there were no significant changes for these markers (Figure S2). To continue with the evaluation of Transfer cells via adrenoreceptors and calcium regulation, adrenergic receptor β3 (Adrβ3) and Sarcoplasmic Reticulum Ca^2^ ^+^ ATPase (SERCA2) were also evaluated. Transfer cells showed an increased Adrβ3 mRNA by day 14 and 21 compared with 32°C differentiated adipocytes, and an intermediate mRNA SERCA2 expression (Figure S3). When samples were analysed by principal component analysis (PCA), adipogenic cells differentiated under normal 37°C culture conditions showed a more defined cluster at later time‐points exhibiting homogeneity in gene expression, while Transfer cells overlapped with both 37°C and 32°C groups (Figure 4D).

**FIGURE 5:**
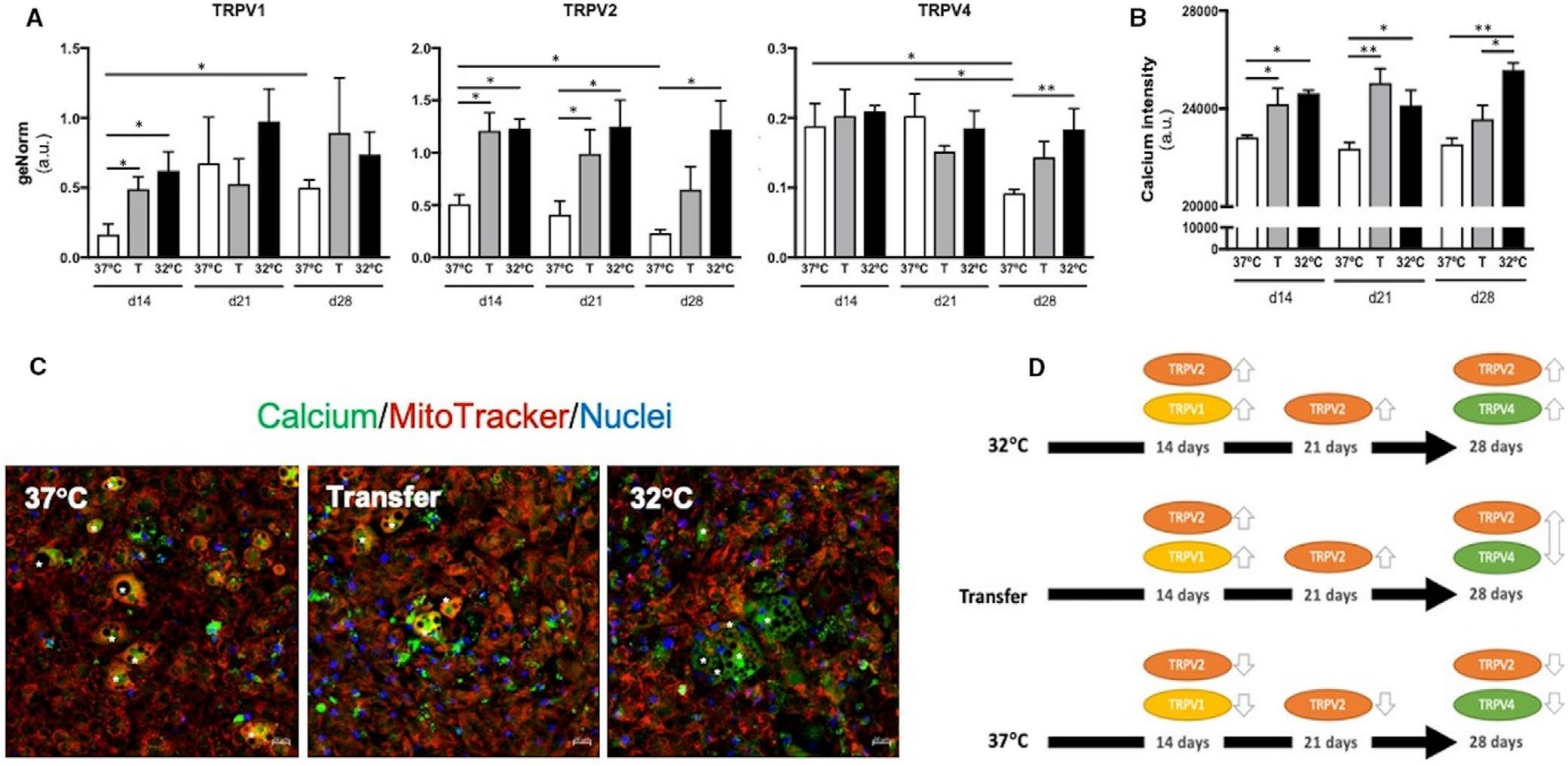
Gene expression analysis of thermotransient receptor potential ion channels. (A) TRPV1, TRPV2 and TRPV4 expression analysed by real‐time PCR. All data were normalized using GeNorm. Data shown as the mean of four replicas ± SEM. Statistical significance was set at *P* < .05. (B) Calcium intensity measurements. Data represent the mean of three replicas ± SEM. Statistical significance was set at *P* < .05. (C) Representative images of calcium staining (Fluo‐4 ‐ green) at day 28. Asterisks indicate differentiated cells showing calcium staining. Transfer image is shown separately in Figure S4 with individual channels. (D) Summary of changes in TRPV expression pattern under the different treatments. Arrow pointing up and down means no significance between 37°C and 32°C treatments

To explore the temperature responsiveness of Transfer adipocytes, gene expression of TRPV ion channels 1, 2 and 4 was evaluated. At day 14, TRPV1 mRNA abundance was increased in Transfer cells and hypothermic conditions compared with normal 37°C conditions, and while TRPV2 maintained a similar trend over the whole time‐course, TRPV4 only showed the same trend at day 28 (Figure 5A). It is known that TRPV activation can increase calcium concentration in cells^21^; hence, intracellular calcium was measured in all time‐points. Cells in Transfer and hypothermic conditions showed higher staining intensity than those under normal culture conditions (Figure 5B, 5C and Figure S4). An overview of changes in TRPV expression pattern under the different treatments can be observed in Figure 5D.

### 3.3 Metabolic function of cold‐induced adipocytes is modified when returned to a thermoneutral environment

**FIGURE 6:**
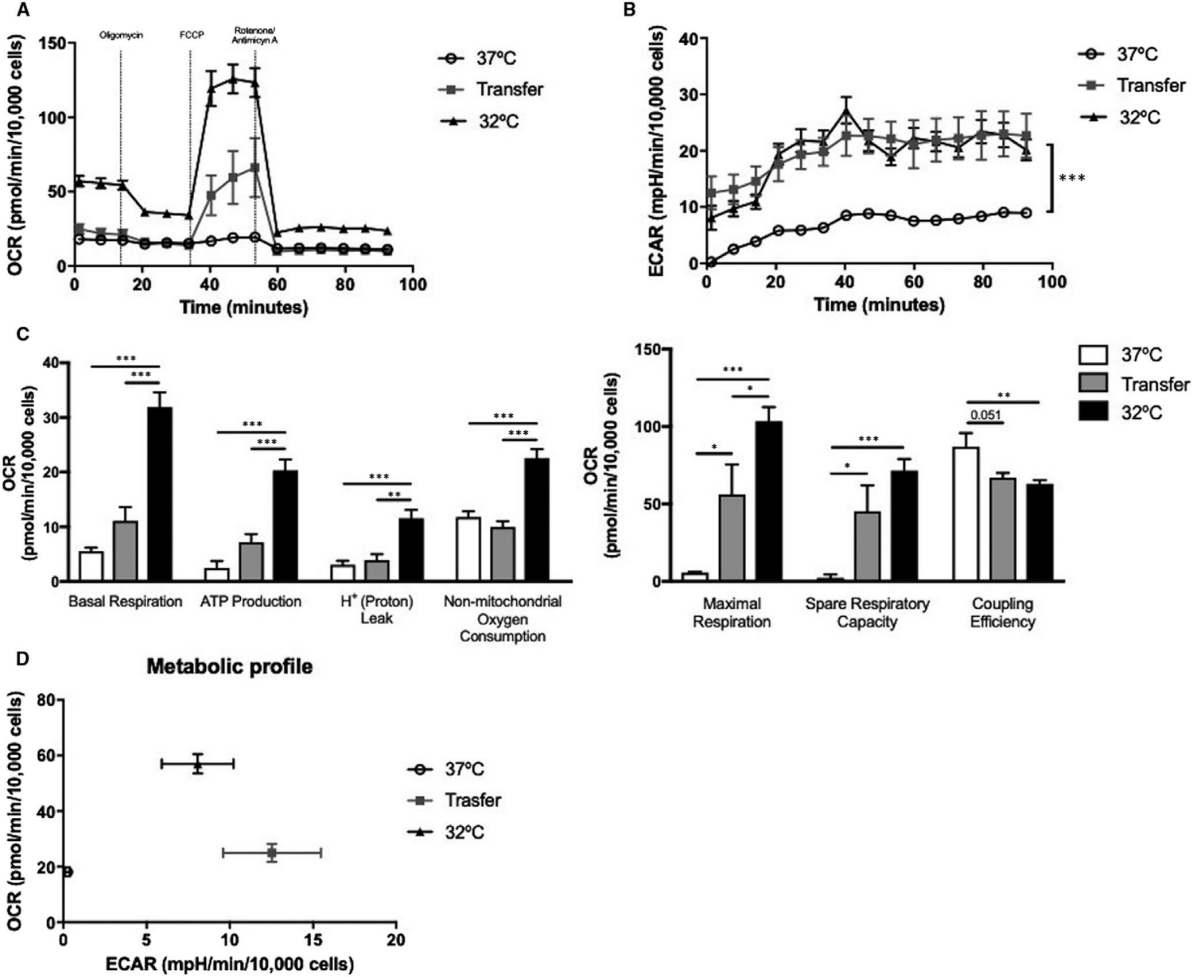
Effect of incubation temperature on the bioenergetics status of cultures after 28 days. (A) Oxygen consumption rate (OCR), (B) Extracellular acidification rate (ECAR), (C) mitochondrial function calculated from the bioenergetics profile (basal respiration, ATP‐linked respiration, proton leak, non‐mitochondrial respiration, maximal respiration, spare respiratory capacity and coupling efficiency) and (D) metabolic profile. Data shown as mean ± SEM, n = 6 individual experiments. *P < .05, **P < .01, ***P < .001

When metabolic function was analysed, the higher rate of oxygen consumption in cells maintained at 32°C was not sustained when transferred to 37°C (Figure 6A). Transfer cells were, however, able to up‐regulate glycolysis to meet the requirement for ATP upon FCCP stimulation (Figure 6B). No differences were observed in basal respiration, ATP production, proton leak and non‐mitochondrial oxygen consumption when Transfer cells were compared with normothermic conditions.

The bioenergetic status of Transfer cells displayed a maximal respiration and spare capacity higher than cells differentiated at 37°C, but significantly lower than cells differentiated at 32°C. Additionally, coupling efficiency of Transfer cells showed no difference between hypothermic 32°C cultures when compared to 37°C (Figure 6C). When plotting basal ECAR against OCR, Transfer adipocytes displayed increased glycolysis and mitochondrial respiration (Figure 6D).

## 4 DISCUSSION

MSCs form adipocytes, and exposure to hypothermic conditions promotes the induction of beige‐like properties, including morphological, biometabolic and molecular traits.^7^, ^10^ The present study investigated whether these beige‐like cells produced in vitro at 32°C were able to adapt to environmental changes when the cells were transferred to normal 37°C culture conditions, and whether their acquired traits may be reversible over time.

### 4.1 The bioenergetics benefits of hypothermic exposure are retained after return to normothermic conditions

Mitochondria evaluation in Transfer adipocytes showed a significant decrease overtime when compared to adipocytes differentiated in hypothermic conditions. Nonetheless, UCP1 protein expression was maintained over the course of the treatment as well as in 32°C adipocytes. These results paired with COX8β mRNA reduction^22^ suggest a decline in mitochondrial content once the browning stimulus is removed, possibly as a step towards whitening.^12^

The bioenergetic analysis further showed that Transfer adipocytes could be activated when the mitochondrial uncoupler FCCP^23^ was added. Higher OCR, ECAR, maximal respiration, spare capacity and metabolic profile than adipocytes differentiated at 37°C reflect a transitional bioenergetic status in which glycolysis rather than mitochondrial respiration was enhanced. Glycolysis has been confirmed to support full adipocyte thermogenesis in BAT by providing pyruvate formation and other intermediates for metabolic pathways as well as maintaining OCR.^24^ In addition, in vivo evidence shows that intracellular glycolysis acts by controlling short‐term non‐shivering thermogenesis.^25^ These results suggest that Transfer cells maintain a high glycolytic rate in order to replenish ATP when uncoupled oxidative phosphorylation and thermogenic activation are occurring.^24^ Together with the mitochondrial measurements and increased UCP1 protein expression^26^ show that Transfer cells are metabolically activated by enhancing lipolysis and fatty acid oxidation, in line with a previous study in 3T3‐L1 adipocytes treated with FCCP.^23^ These observations also suggest that MSC‐derived beige adipocytes demonstrate a distinctive bioenergetic response, as they can both store lipids and produce heat when stimulated,^27^ adding to previous *in vitro* ^7^, ^10^ and *in vivo* ^28^, ^29^ reports to highlight MSCs as a valuable resource to study brown/beige adipogenesis.

### 4.2 The induced beige‐like phenotype is not fully reversible

When evaluating the phenotype of Transfer cells, they exhibited an intermediary amount of lipid content, with a morphology and percentage of differentiated cells more similarly to cells kept at 32°C than cells differentiated under normothermic culture conditions. This is in accord with the two main processes of brown adipocyte generation, that is de novo and trans‐differentiation.^30^ Additionally, lipogenesis and lipolysis, two important mechanisms in brown adipocytes, are required for UCP1 proton transport^31^, ^32^ and could explain why adipocytes differentiated in hypothermic and in Transfer conditions contained more lipid and differentiated cells. Even though Transfer cells possessed less mitochondria than hypothermic differentiated adipocytes, their ability to retain UCP1 protein could be due to the capacity to maintain a brown/beige morphology with multilocular LDs, which provided more substrate availability than possessing larger or unilocular LDs.

### 4.3 Beige‐brown marker expression

Following reports that human BAT may be essentially comprised by beige adipocytes,^33^ the activation of beige marker gene expression has raised much interest; however, the stability of these characteristics remains unclear. Transfer adipocytes were observed here to acquire a transitional pattern for mRNA expression. A decrease in AdipoQ mRNA was observed, a marker which has been directly linked to obesity^34^; however, maintenance of FABP4 and PPARγ suggested retention of a brown‐like signature as these two genes play an important role in brown adipocyte physiology and function.^35^, ^36^ UCP1 expression is present in brown and beige adipocytes, to uncouple respiration and release energy in the form of heat^37^, ^38^, ^39^, ^40^, ^41^; however, recent studies report that beige transition to white occurs after 15 days of stimuli withdrawal. These cells thus lose their brown characteristics, specifically UCP1 mRNA and protein signatures.^7^, ^12^ Here, Transfer cells showed a similar UCP1 mRNA and protein expression to 32°C differentiated adipocytes after 3 weeks of acclimation, demonstrating their ability to retain an uncoupling capacity after being returned to warm conditions. The intermediary COX8β mRNA expression of Transfer cells could be indicative of their response to stress under conditions of increase energy demand, since COX8β is involved in respiratory electron transport and marks beigeing in mouse WAT.^42^ However, the decrease in UCP1 and COX8β after 3 weeks, paired with an intermediate bioenergetic profile, enhanced glycolysis and high intracellular calcium intensity in Transfer cells could suggest a shared mechanism of calcium‐mediated UCP1‐independent thermogenesis.^43^ Furthermore, high expression of the classical beige and brown adipogenic markers CIDEA^42^ and LHX8^44^, ^45^, ^46^ were detected in Transfer cells. CIDEA plays a major role in controlling lipid storage, lipid droplet fusion^47^, ^48^ and uncoupling UCP1 activity in BAT upon cold exposure.^42^ Here, its high expression could be involved in maintaining the intermediate morphology in Transfer adipocytes^49^ by mediating the growth of LDs upon acclimatization to normal culture conditions and controlling lipogenesis and lipolysis.^48^ Moreover, the substantial increase in CIDEA in Transfer cells is in line with previous data showing that besides UCP1 and LHX8, CIDEA represents the best marker to evaluate the beigeing of WAT during cold treatment.^42^

Taken together, these results suggest that the molecular signature of Transfer adipocytes is distinct from that of cells differentiated under constant 37°C or 32°C conditions. Our results agree to an extent with that reported by Altshuller‐Keylin and colleagues, describing a full transition of beige to white adipocytes lasting 15‐20 days.^12^ Here, an intermediate beige molecular signature was still present 3 weeks after adipocytes were transferred back to 37°C, highlighting the plasticity of MSCs to generate and preserve de novo beige adipocytes. Moreover, the intermediate profile of Transfer MSCs resembles to what Barbatelli and colleagues described before as UCP1 + pauci‐locular adipocytes, located in mouse WAT after cold acclimatization.^30^

### 4.4 Temperature sensing channels

This study revealed a complex interaction between TRPV1, 2 and 4 over the course of the differentiation treatments. TRPV1 increases when cold stimulation is applied to adipocytes,^7^, ^10^ and its activation at a pre‐adipocyte stage could be clinically beneficial by increasing intracellular calcium levels and preventing adipogenesis.^50^ Furthermore, TRPV2 mRNA expression is known to increase in differentiated mouse brown adipocytes.^51^, ^52^ Here, it could be that a cascade of TRPV channels is necessary to retain the bioenergetics response, starting with the activation of TRPV1, maintenance of TRPV2 and finally the activation of TRPV4. Sun and colleagues showed that TRPV2 is not only the highest expressed thermoreceptor channel member, but also essential in brown adipocytes thermogenic function.^51^, ^52^, ^53^ Taking into account the importance of calcium in UCP1‐dependent^54^ and UCP1‐independent adipocyte thermogenesis,^43^, ^54^, ^55^ it is possible that TRPV2 is one of the main regulators for beigeing maintenance and/or temperature sensing mechanism of cells.^52^ Although the role of TRPV4 is debated,^56^, ^57^ by day 28 Transfer cells displayed a pattern of TRPV4 and UCP1 mRNA expression similar to 32°C adipocytes. These results, together with the bioenergetics response, could indicate a positive correlation with TRPV4 intermediate expression and browning. This observation supports its role as one regulator of BAT oxidative metabolism.^58^ Complementary targeted experiments including the modulation of TRPV activation and in particular TRPV2 could confirm the mechanism involved and provide a potential strategy to modulate obesity and related disorders.

Overall, these results suggest that MSCs differentiated under Transfer conditions retain some beige‐like features rather than completely reverting to a white phenotype, even after an extended incubation in normal temperature culture conditions of 3 weeks. The results presented here not only confirm that stem cells can autonomously respond and phenotypically adapt to temperature conditions, but also demonstrate that once beige cell characteristics are acquired, they are not fully reversible. This suggests a type of cellular imprint reflecting prior environmental culture conditions. It could thus be useful to monitor the epigenetic status of differentiating MSCs upon change in temperature conditions. These results also underline MSCs as versatile and accessible cell source to model the differentiation and maintenance of beige adipocytes.

## 5 CONCLUSION & PERSPECTIVES

The increasing drive to develop and maintain brown fat in humans, due to the possible associated health benefits, calls for robust preclinical models to be established. Using MSCs, our results show beige‐like traits induced under hypothermic conditions are retained even after cells are returned to a standard temperature. Further experiments could now be undertaken to investigate whether the maintenance of beigeing features can similarly be observed in human adipogenic cultures. The persistence of these cellular features suggests cell responses to environmental changes are influenced by a mechanism of differentiation memory, which requires further investigation.

The in vivo behaviour of such conditioned adipocytes remains to be evaluated in order to determine whether their browning properties may be retained upon transplantation into recipient tissue. If confirmed in vivo, this prolonged maintenance of brown/beige adipocytes might provide an experimental approach to promote energy expenditure, possibly combined with additional browning stimuli, which may be relevant for obesity research.

## CONFLICT OF INTEREST

The authors declare no conflict of interest regarding the publication of this manuscript.

## **AUTHOR** **CONTRIBUTIONS**

HALL, MS and VS: Project plan and design. KV and HALL: Assembling the figure and supporting information. KV and HALL: Cellular experiments and data analysis. HALL and IB: Molecular analysis. HALL, MS and VS: Manuscript finalization. All authors: Scientific review and discussion of the manuscript.

## Supporting information

Fig S1‐4

## Data Availability

The data that support the findings of this study are available from the corresponding author upon reasonable request.
