# Supplementary material for: Cold‐induced beigeing of stem cell‐derived adipocytes is not fully reversible after return to normothermia
Source: J Cell Mol Med. 2020 Sep 9;24(19):11434–44. doi: 10.1111/jcmm.15749 (PMC7576274; doi:10.1111/jcmm.15749)
Supplement: Supplementary file 1 — Fig S1‐4 [file JCMM-24-11434-s001.pdf]

Cold-induced beigeing of stem cell-derived adipocytes is not fully reversible after return to normothermia.

Hilda Anaid Lugo Leija[1], Ksenija Velickovic[1], Ian Bloor[3], Harold Sacks[2], Michael E. Symonds[3,4], Virginie Sottile[1,5*].

[1] Wolfson STEM Centre, School of Medicine, The University of Nottingham, UK
[2] VA Endocrinology and Diabetes Division, Department of Medicine, University of California, Los Angeles, USA
[3] The Early Life Research Unit, Division of Child Health, Obstetrics and Gynaecology, and [4]Nottingham Digestive Disease Centre and Biomedical Research Centre, School of Medicine, The University of Nottingham, UK
[5] Department of Molecular Medicine, The University of Pavia, Pavia, Italy.

* Correspondence to virginie.sottile@unipv.it

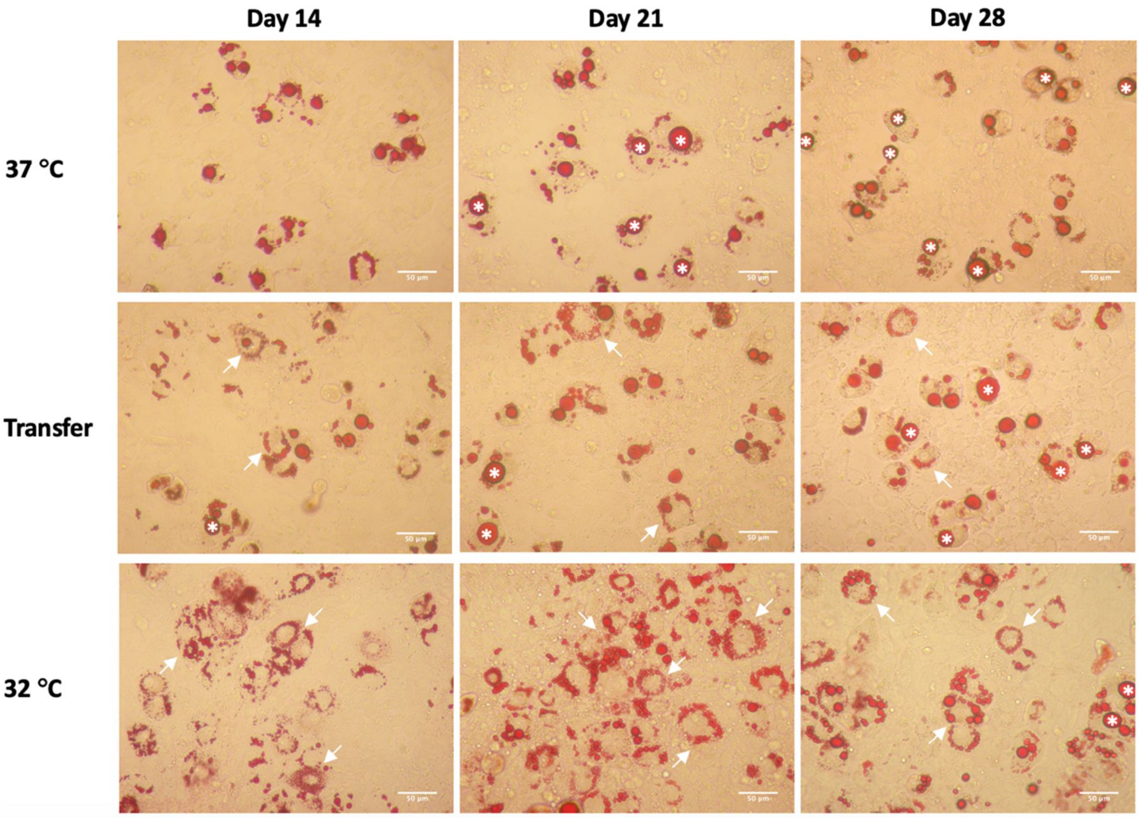

Supplementary Fig. 1 Oil Red O (ORO) staining of mMSCs differentiated under adipogenic treatment in normal, transferred and hypothermic conditions. Representative images of ORO at day 14, 21 and 28 of differentiation treatment. Arrows indicate multilocular cells and asterisks indicate cells with one single lipid droplet

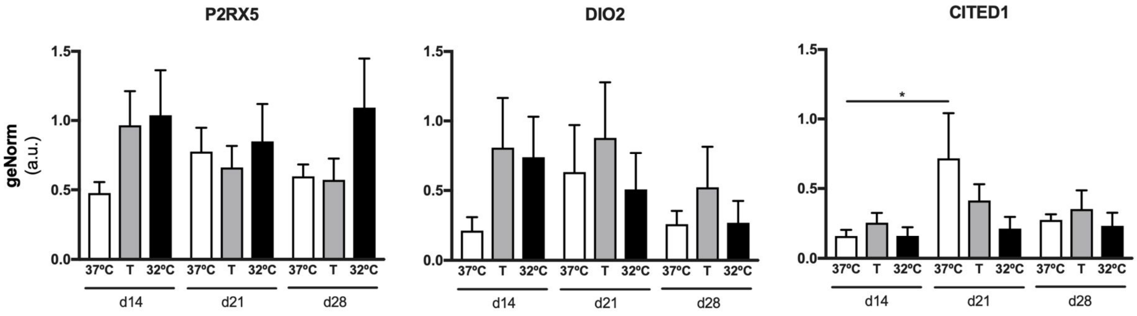

Supplementary Fig. 2 Gene expression analysis of brown/beige markers. Expression of brown and beige adipogenic markers P2RX5, DIO2 and CITED1. All data was normalised using GeNorm. Data are shown as mean ± SEM. *p<0.05, **p<0.01, ***p<0.001

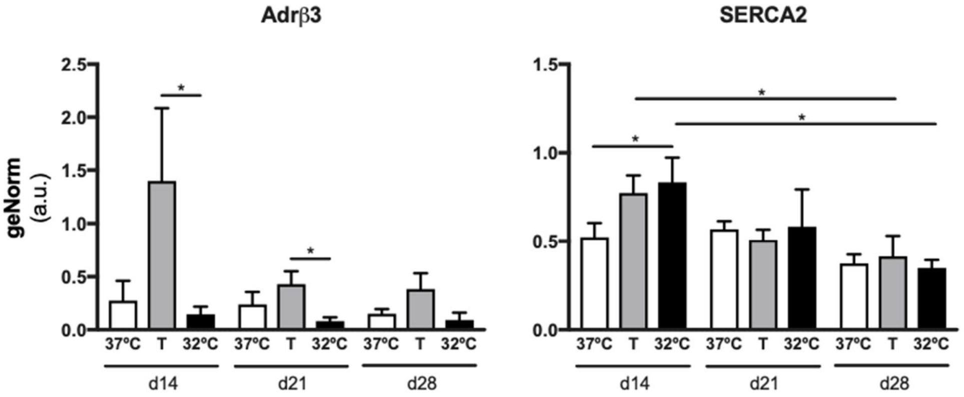

Supplementary Fig. 3 Gene expression analysis of Adrenergic receptor β3 (Adrβ3) and Sarcoplasmic Reticulum Ca^{2+} ATPase (SERCA2). All data was normalised using GeNorm. Data are shown as mean ± SEM. *p<0.05, **p<0.01, ***p<0.001

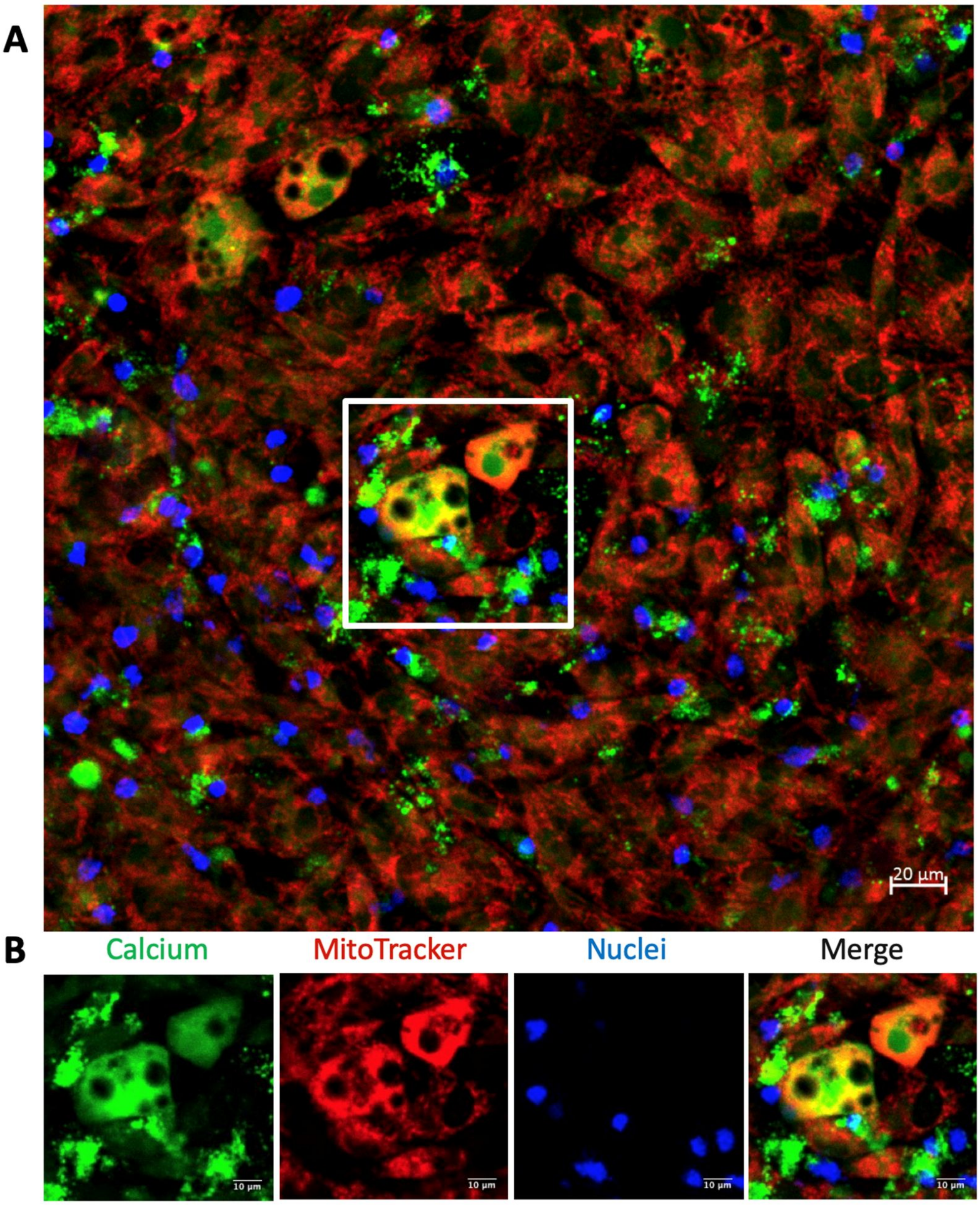

Supplementary Fig. 4 Live staining showing calcium staining in Transfer cells at day 28 (enlarged image of Figure 5C). (a) Representative image showing the prevalence of calcium staining (Fluo-4 Direct in green), MitoTracker (red), Hoechst 33258 was used to identify nuclei (blue). The dashed square highlights difference in staining between two adjacent adipocytes. Scale bar: 20 µm. (b) Enlarged view of the square area, with individual and merged images showing adipocytes positive for both calcium (green) and MitoTracker (red). Scale bars: 10µm. n = 3 individual experiments
